# An antibody-escape estimator for mutations to the SARS-CoV-2 receptor-binding domain

**DOI:** 10.1093/ve/veac021

**Published:** 2022-05-11

**Authors:** Allison J Greaney, Tyler N Starr, Jesse D Bloom

**Affiliations:** Department of Genome Sciences, University of Washington, 3720 15th Ave NE, Seattle, WA, USA; Medical Scientist Training Program, University of Washington, 3720 15th Ave NE, Seattle, WA, USA; Basic Sciences and Computational Biology, Fred Hutchinson Cancer Center, 1100 Fairview Ave N, Seattle, WA, USA; Howard Hughes Medical Institute Seattle, 1100 Fairview Ave N, Seattle, WA, USA; Basic Sciences and Computational Biology, Fred Hutchinson Cancer Center, 1100 Fairview Ave N, Seattle, WA, USA; Department of Genome Sciences, University of Washington, 3720 15th Ave NE, Seattle, WA, USA; Howard Hughes Medical Institute Seattle, 1100 Fairview Ave N, Seattle, WA, USA

**Keywords:** deep mutational scanning, antibody escape, epitope, SARS-CoV-2 variants, Omicron

## Abstract

A key goal of severe acute respiratory syndrome coronavirus 2 (SARS-CoV-2) surveillance is to rapidly identify viral variants with mutations that reduce neutralization by polyclonal antibodies elicited by vaccination or infection. Unfortunately, direct experimental characterization of new viral variants lags their sequence-based identification. Here we help address this challenge by aggregating deep mutational scanning data into an ‘escape estimator’ that estimates the antigenic effects of arbitrary combinations of mutations to the virus’s spike receptor-binding domain. The estimator can be used to intuitively visualize how mutations impact polyclonal antibody recognition and score the expected antigenic effect of combinations of mutations. These scores correlate with neutralization assays performed on SARS-CoV-2 variants and emphasize the ominous antigenic properties of the recently described Omicron variant. An interactive version of the estimator is at https://jbloomlab.github.io/SARS2_RBD_Ab_escape_maps/escape-calc/ (last accessed 11 March 2022), and we provide a Python module for batch processing. Currently the calculator uses primarily data for antibodies elicited by Wuhan-Hu-1-like vaccination or infection and so is expected to work best for calculating escape from such immunity for mutations relative to early SARS-CoV-2 strains.

Human coronaviruses undergo antigenic evolution that erodes antibody-based neutralization (Eguia et al., 2021; Kistler and Bedford, 2021). This antigenic evolution is already apparent for severe acute respiratory syndrome coronavirus 2 (SARS-CoV-2), as new viral variants with reduced antibody neutralization emerged within a year of when the virus first started to spread in humans. A tremendous amount of experimental effort has been expended to characterize these SARS-CoV-2 variants in neutralization assays (Lucas et al., 2021; Uriu et al., 2021; Wang et al., 2021). Unfortunately, the rate at which new variants arise outstrips the speed at which these experiments can be performed.

A partial solution is to use deep mutational scanning experiments to *prospectively* measure how viral mutations impact antibody binding or neutralization. Deep mutational scanning can systematically measure the antigenic impacts of all possible amino-acid mutations in the key regions of spike on monoclonal antibodies (Starr et al., 2021b; Greaney et al., 2021c) or sera (Greaney et al., 2021a). However, SARS-CoV-2 variants of concern (variants with reduced immune recognition, enhanced transmissibility, or increased virulence) typically have multiple mutations, and it is not feasible to experimentally characterize all combinations of mutations even via high-throughput approaches such as deep mutational scanning.

Here we take a step toward addressing this challenge by aggregating deep mutational scanning data across many antibodies to assess the impacts of mutations in the spike receptor-binding domain (RBD), which is the primary target of neutralizing antibodies to SARS-CoV-2 (Greaney et al., 2021a; Piccoli et al., 2020; Schmidt et al., 2021). The resulting ‘escape estimator’ enables qualitative visualization and quantitative scoring of the antigenic effects of arbitrary combinations of mutations. Importantly, the escape estimator is based on simple transformations of direct experimental measurements, and so its calculations can be intuitively visualized using the interactive interface we provide.

## 1. Results

### 1.1. Combining monoclonal antibody-escape maps reveals correlated and independent viral antigenic mutations

A deep mutational scanning experiment can measure how all single amino-acid mutations to the SARS-CoV-2 RBD affect binding by a monoclonal antibody (Greaney et al., 2021c). This mutation-level information can be summarized for each RBD site, such as by taking the mean or sum of mutation-level effects at a site. Here we will work with these site-level escape maps. We use site-level information for two reasons: mutation-level measurements tend to be more noisy and averaging them for a site decreases this noise, and using site-level information makes the approach independent of the particular wild-type amino acid at a site (which is useful if we want to keep using the calculator as the RBD evolves). However, we note that site-level information ignores the possibility of different mutations at a site having different effects, and so mutation-level approaches could also become useful as the quality of experimental data improves.

**Figure 1.**
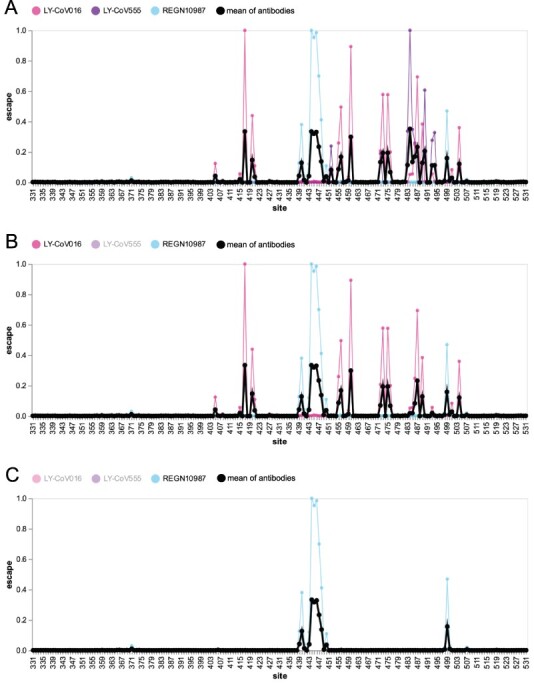
Escape map for a hypothetical polyclonal mix consisting of an equipotent mixture of three monoclonal antibodies targeting distinct epitopes on the SARS-CoV-2 RBD. (A) Experimentally measured escape maps for three antibodies, and the mean of these maps (thick black line). Each point on the *x*-axis represents a site in the RBD, and the *y*-axis represents the total measured escape by all mutations at that site scaled so the maximum for each antibody is one. (B) Escape map if the contribution of antibody LY-CoV555 is ablated. (C) Escape map if the contributions of antibodies LY-CoV555 and LY-CoV016 are ablated. An interactive version of this figure is at https://jbloomlab.github.io/SARS2_RBD_Ab_escape_maps/mini-example-escape-calc/ (last accessed 11 March 2022).

As a small example to illustrate the principle behind our approach, Fig. 1A shows previously reported measurements (Starr et al., 2021b, c) of how mutations to each RBD site affect binding by three monoclonal antibodies: LY-CoV016 (etesevimab), LY-CoV555 (bamlanivimab), and REGN10987 (imdevimab). Each antibody targets a different epitope on the RBD: LY-CoV016 targets the Class 1 epitope, LY-CoV555 the Class 2 epitope, and REGN10987 the Class 3 epitope (Barnes et al., 2020; Greaney et al., 2021b). Because the antibodies have distinct epitopes, they are escaped by largely distinct sets of mutations: LY-CoV016 is most strongly escaped by mutations at Site 417, LY-CoV555 at Site 484, and REGN10987 at Sites 444–446 (Fig. 1A).

Now consider a hypothetical polyclonal antibody mix of these three antibodies combined at equal potencies. We can generate an escape map for this hypothetical antibody mix simply by averaging the experimentally measured escape maps for the three individual antibodies, yielding the thick black line in Fig. 1A. Because this polyclonal escape map is the average of the monoclonal antibody maps, its largest peaks are at the sites of strongest escape for each individual antibody: 417, 484, and 444–446.

Next consider removing one antibody from the hypothetical mix by mutating its epitope. Figure 1B shows the resulting escape map if LY-CoV555 is ablated, as would occur if Site 484 was mutated. The thick black line for the antibody mix no longer has peaks at 484 and other sites targeted by LY-CoV555, such as 490. Therefore, in this hypothetical polyclonal antibody mix, escape at Sites 484 and 490 is correlated since both sites are targeted by the same antibody. However, the polyclonal mix’s escape map at Sites 417 and 460 is unaffected by mutations that escape LY-CoV555, since they are targeted by a different antibody, LY-CoV016. But if we also ablate LY-CoV016 (such as by mutating Site 417), then the peaks at 417 and 460 also disappear, and the remaining peaks are at sites targeted by REGN10987, such as 444–446 (Fig. 1C). Of course, if REGN10987 was also ablated such as by mutating Site 446, then the polyclonal antibody mix would have no remaining activity. This and other scenarios can be explored using the interactive version of Fig. 1 at https://jbloomlab.github.io/SARS2_RBD_Ab_escape_maps/mini-example-escape-calc/ (last accessed 11 March 2022).

### 1.2. Aggregating deep mutational scanning data for thirty-three human antibodies yields a realistic escape estimator

The illustrative example in the previous section illustrates how experimental data for individual antibodies can be combined to yield an escape map for a hypothetical polyclonal antibody mix. To create an escape map for an antibody mix that more realistically represents the actual human sera, we aggregated previously generated deep mutational scanning data for thirty-three neutralizing antibodies elicited by SARS-CoV-2. These antibodies were isolated from a variety of patient cohorts within the first year of the pandemic (see Methods for details). An assumption of the analysis that follows is that an equipotent mixture of these thirty-three antibodies represents the neutralizing activity of human sera; we emphasize that this assumption is imperfect since in reality the antibodies were chosen for prior study for a variety of ad hoc reasons. The escape maps for all the individual antibodies can be interactively interrogated at https://jbloomlab.github.io/SARS2_RBD_Ab_escape_maps/ (last accessed 11 March 2022).

**Figure 2.**
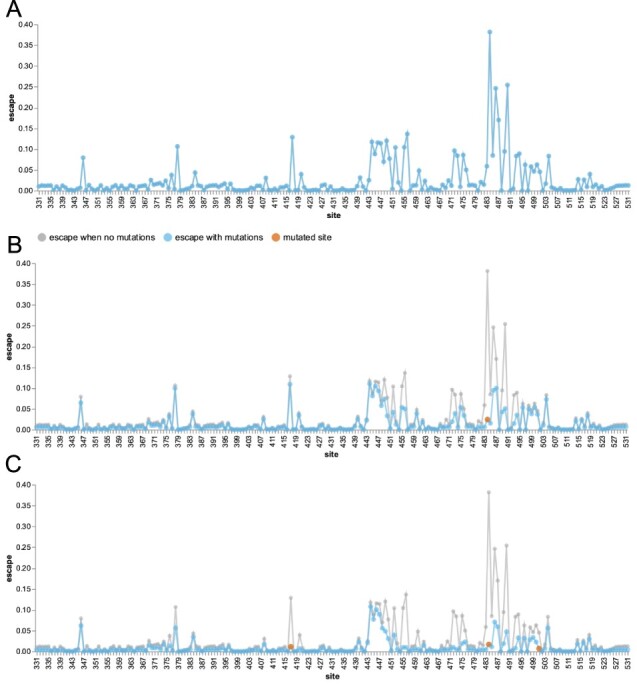
An escape estimator generated by aggregating deep mutational scanning for thirty-three neutralizing antibodies targeting the SARS-CoV-2 RBD. (A) The blue line shows the extent of escape mediated by mutations at each site, as estimated by simply averaging the data for all the individual antibodies. (B) The blue line shows escape map after a mutation to Site 484 (red point) ablates recognition by antibodies strongly targeting that site, while the gray line shows the original escape map in the absence of any mutations. (C) The escape map after mutating Sites 417, 484, and 501 (the three RBD sites mutated in the Beta variant). An interactive version of this figure is at https://jbloomlab.github.io/SARS2_RBD_Ab_escape_maps/escape-calc/ (last accessed 11 March 2022).

The overall polyclonal escape map generated by averaging the experimental data for all thirty-three antibodies is in Fig. 2A. As in the illustrative three-antibody example in the previous section, there are peaks at Sites 417, 484, and 444–446. However, the peak at 484 is now larger than any other peak, reflecting the fact that antibodies targeting the Class 2 epitope containing E484 are especially common in the human antibody response to early SARS-CoV-2 strains (Chen et al., 2021; Greaney et al., 2021a, b; Robbiani et al., 2020; Yuan et al., 2020). In addition, there are smaller peaks at a variety of other sites, reflecting the fact that each antibody has a somewhat idiosyncratic epitope (Fig. 2A).

We can follow the principle outlined in the illustrative example in the previous section to estimate the expected polyclonal escape map *after* mutating sites in the RBD. Specifically, we reduce the contribution of each antibody by an amount that scales with how strongly that antibody targets each mutated site (see Methods for details). For instance, the blue lines in Fig. 2B show the polyclonal escape map after mutating Site 484. Mutating Site 484 obviously drops the contribution of that site, but it also decreases the contribution of other sites such as 490 that are commonly targeted by antibodies with epitopes that include Site 484. In contrast, mutating Site 484 has minimal effect on the polyclonal escape map at sites like 417 or 444–446, since those sites are generally targeted by antibodies that are unaffected by mutations at Site 484.

We can also calculate the expected effects of compound mutations. Figure 2C shows the polyclonal escape map after mutating all three RBD sites that are changed in the Beta variant (Sites 417, 484, and 501). This polyclonal escape map has lost contributions not only from the mutated sites, but also sites that form common epitopes with 417 or 484 (e.g., Sites 455, 456, 486, and 490). However, the escape map still has major contributions from antibodies targeting sites like 444–446, since such antibodies are generally unaffected by mutations at Sites 417, 484, and 501.

We recommend the reader explore the interactive escape estimator at https://jbloomlab.github.io/SARS2_RBD_Ab_escape_maps/escape-calc/ (last accessed 11 March 2022), to perform calculations like those in Fig. 2 for arbitrary combinations of mutated RBD sites. Such visual exploration of different combinations of mutations provides an intuitive sense of the antigenic structure of the RBD.

### 1.3. The escape calculations correlate well with neutralization assays of human polyclonal sera against SARS-CoV-2 variants

For each set of mutated RBD sites, we can define a quantitative score that represents the polyclonal antibody binding that remains after mutating these sites. This score is defined using the same principle as the site-wise escape estimator in the previous section: we reduce the contribution of each antibody by an amount that scales with how strongly it is escaped by each mutated site and define the overall score as the fraction of all antibody contributions that remain (see Methods for details). This calculation is implemented in the interactive estimator at https://jbloomlab.github.io/SARS2_RBD_Ab_escape_maps/escape-calc/ (last accessed 11 March 2022) and returns a score that ranges from one (no mutations affect binding of any antibodies) to zero (all antibodies fully escaped).

**Figure 3.**
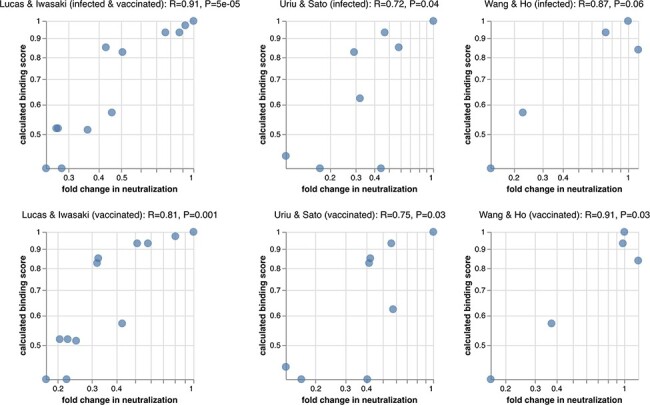
Correlation of calculated binding scores with experimentally measured fold changes in neutralization for SARS-CoV-2 variants and mutants (smaller values indicate worse neutralization). The data of Lucas et al. (2021) were generated using authentic SARS-CoV-2 and sera from vaccinated individuals who were (top) or were not (bottom) previously infected with SARS-CoV-2. The data of Uriu et al. (2021) and Wang et al. (2021) were generated using pseudovirus against convalescent (top) or vaccine (bottom) sera, with vaccine sera from Pfizer BNT162b2 or Moderna mRNA-1273 vaccines, respectively. The fold changes are geometric means over all subjects in each cohort. Labels at the top of each plot show the Pearson’s R and associated *P*-value. An interactive version of this figure that allows mousing over points to see details is at https://jbloomlab.github.io/RBD_escape_calculator_paper/neut_studies.html (last accessed 11 March 2022). Supplementary Fig. S1 shows almost identical results are obtained if the binding scores are computed using the mean of mutations at a site rather than the sum.

To test how these escape-estimator scores compare to experimentally measured neutralization titers, we collated neutralization data from three previously published studies (Lucas et al., 2021; Uriu et al., 2021; Wang et al., 2021), each of which characterized sera from two patient cohorts against a variety of SARS-CoV-2 variants and mutants. One can imagine many reasons why the escape-estimator scores might differ from the real neutralization titers: the estimator only considers RBD mutations, the antibodies used by the estimator might not accurately reflect the real mix in polyclonal sera, etc. But despite all these potential caveats, the escape-estimator scores correlate quite well with the measured neutralization titers across all studies and cohorts (Fig. 3). Therefore, the simple and intuitive approach used by the estimator seems to accurately reflect the dominant features of polyclonal antibody escape in the RBD.

### 1.4. The escape estimator suggests extensive antigenic change in the new Omicron variant

**Figure 4.**
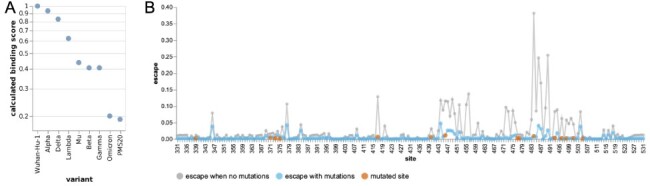
Escape calculations for the Omicron variant. (A) The calculated binding scores for SARS-CoV-2 variants and the artificial polymutant spike (PMS20) generated by Schmidt et al. (2021). Scores of one indicate no mutations affect binding, and scores of zero indicate no antibody binding remains. An interactive version of this plot that allows mousing over points to see details is at https://jbloomlab.github.io/RBD_escape_calculator_paper/variants.html (last accessed March-11-2022). (B) The calculated escape map for the Omicron variant’s RBD (blue) compared to an unmutated RBD (gray), with sites of mutations in the Omicron variant in red. The mutated RBD sites for each variant are in Table 1.

We applied the escape estimator to the recently reported Omicron variant, which has fifteen mutated sites in its RBD (de Oliveira, 2021; NGS-SA, 2021). The calculated binding score for the Omicron variant is much lower than any other SARS-CoV-2 variants of concern, indicating extensive antibody escape (Fig. 4A). The Omicron variant’s calculated score is roughly equivalent to that of a polymutant spike (PMS20) that was artificially engineered in a pseudovirus by Schmidt et al. (2021) to maximize escape from polyclonal serum antibodies. For comparison, Schmidt et al. (2021) measured that neutralization titers against this artificial PMS20 spike were reduced by ~20- to ~80-fold for sera from various cohorts of vaccinated and infected individuals.

**Table 1.** Mutated RBD sites in SARS-CoV-2 variants.

Variant	Mutated RBD sites
Wuhan-Hu-1	
Alpha	501
Beta	417 484 501
Gamma	417 484 501
Delta	452 478
Lambda	452 490
Mu	346 484 501
Omicron	339 371 373 375 417 440 446 477 478 484 493 496 498 501 505
PMS20	346 417 440 445 455 475 484 501

The site-level escape map for the Omicron variant’s RBD is shown in Fig. 4B. The Omicron RBD has lost most peaks of antibody binding relative to the original RBD. Exploration of the mutations using the interactive estimator at https://jbloomlab.github.io/SARS2_RBD_Ab_escape_maps/escape-calc/ (last accessed 11 March 2022) indicates that mutations at Sites 484, 446, and 417 are the largest drivers of this antigenic change, although other mutations also contribute. The residual peaks in the map suggest that the remaining antibody activity against the Omicron variant RBD could be further eroded by mutations at sites like 346, 378, 444, and 504.

## 2. Discussion

We have described an escape estimator that uses experimental data for thirty-three monoclonal antibodies to estimate the antigenic effects of arbitrary combinations of mutations to the SARS-CoV-2 RBD. The key insight is to aggregate data for individual antibodies to define both which RBD sites are antigenically important and which combinations of mutations have redundant versus additive effects on antibody binding. For instance, Sites 417, 484, and 490 are all peaks of antibody escape. But mutations at 484 and 490 have redundant effects since they generally escape the same antibodies, whereas mutations at 484 and 417 have additive effects since they generally escape different antibodies.

Another key aspect of our approach is the interactive visual implementation of the escape estimator, so the user can interrogate the effects of mutations (or combinations of mutations) simply by clicking (or shift-clicking) on sites. This interactivity provides an intuitive understanding of the antigenic structure of the RBD and shows how the estimator is performing simple transformations directly on experimental data. We encourage interactive use of the estimator so it acts as a visual aid to augment human interpretation. However, we note that in addition to the interactive estimator at https://jbloomlab.github.io/SARS2_RBD_Ab_escape_maps/escape-calc/ (last accessed 11 March 2022), we also provide a Python module for batch processing of large numbers of sequences at https://github.com/jbloomlab/SARS2_RBD_Ab_escape_maps/ (last accessed 11 March 2022).

There are caveats that should be kept in mind when using the escape estimator. First, the estimator only considers sites in the RBD and ignores mutations to other regions of spike. Second, the estimator assumes that the neutralizing activity of human polyclonal serum is represented by an equipotent mix of the monoclonal antibodies that happen to have been previously characterized by deep mutational scanning. Third, the estimator simply averages site-level escape measurements across antibodies and ignores differences in the effects of different amino-acid mutations at the same site. Fourth, the estimator does not yet implement a real biophysical model of the combined activity of multiple antibodies (Einav and Bloom, 2020). Finally, and in our minds most significantly, the estimator estimates the impact of mutations in reference to antibodies targeted to the early Wuhan-Hu-1 RBD—an approach that is currently reasonable, but will become problematic as human exposure and vaccination histories diversify in the years to come (see the last paragraph).

Despite all these caveats, the escape estimator yields binding scores that correlate with experimentally measured neutralization titers. In addition, the actual antigenic evolution of SARS-CoV-2 seems to follow the principles captured by the escape estimator: variants of concern generally have combinations of mutations calculated to have additive effects on antibody escape (e.g., 417 and 484) rather than combinations calculated to have redundant effects (e.g., 484 and 490). We suspect the estimator works well because the RBD is the dominant target of neutralizing activity (Greaney et al., 2021a; Piccoli et al., 2020; Schmidt et al., 2021) and the human antibody response to the early Wuhan-Hu-1 RBD shares broad commonalities across individuals (Chen et al., 2021; Greaney et al., 2021a, b; Robbiani et al., 2020; Yuan et al., 2020).

However, the situation will become more complex over time. Currently, most humans with antibodies to SARS-CoV-2 have been exposed to an RBD antigen that is identical or very similar to that of the early Wuhan-Hu-1 strain. Therefore, antigenic studies can reasonably define mutations in reference to that RBD, since it is what the antibodies target. But as humans are exposed to more diverged RBD variants, it will become difficult to determine what reference to use to define antigenic mutations, since different individuals will have antibodies targeting different RBDs. Additionally, differing exposure histories can leave individuals with different antibody specificities (Cobey and Hensley, 2017), a process that is already starting to occur for SARS-CoV-2 (Greaney et al., 2022). So in the future, it will be necessary to stratify the data used by the escape estimator by which RBD variant elicited the antibodies and aggregate data for antibodies that reflect the sera in question. For this reason, we expect to continue adding to the data used by the escape estimator and emphasize that it will change over time from the version described here, although we provide stable links to the current version in the Methods section.

## 3. Methods

### 3.1. Code and data availability

The most up-to-date code and data used to implement the escape estimator are at https://github.com/jbloomlab/SARS2_RBD_Ab_escape_maps (last accessed 11 March 2022), and the version described in this paper is at https://github.com/jbloomlab/SARS2_RBD_Ab_escape_maps/tree/bioRxiv_v1 (last accessed 11 March 2022).

The data used by the escape estimator are at https://github.com/jbloomlab/SARS2_RBD_Ab_escape_maps/blob/main/processed_data/escape_calculator_data.csv (last accessed 11 March 2022), and the version used for this paper is at https://github.com/jbloomlab/SARS2_RBD_Ab_escape_maps/blob/bioRxiv_v1/processed_data/escape_calculator_data.csv (last accessed 11 March 2022).

A downloadable HTML version of the escape estimator is at https://github.com/jbloomlab/SARS2_RBD_Ab_escape_maps/raw/main/docs/_includes/escape_calc_chart.html (last accessed 11 March 2022), with the version described in this paper at https://github.com/jbloomlab/SARS2_RBD_Ab_escape_maps/blob/bioRxiv_v1/docs/_includes/escape_calc_chart.html (last accessed 11 March 2022).

### 3.2. Interactive versions of figures

Interactive versions of all of the figures in this paper are at https://jbloomlab.github.io/RBD_escape_calculator_paper/ (last accessed 11 March 2022). These figures allow mousing over points to see details, etc.

### 3.3. Deep mutational scanning data used by the estimator

The experimental data used by the escape estimator are drawn from seven previously published deep mutational scanning studies (Dong et al., 2021; Greaney et al., 2021c, b; Starr et al., 2021a, b; c; Tortorici et al., 2021) and one unpublished dataset available at https://github.com/jbloomlab/SARS-CoV-2-RBD_MAP_COV2-2955 (last accessed 11 March 2022). In total, these studies contain data for thirty-six monoclonal antibodies. Three of these antibodies (CR3022, S304, and S309) were elicited by infection with SARS-CoV-1 and so are excluded from the datasets used for the calculations in this paper, although the estimator has an option (eliciting_virus) that allows optional inclusion of these antibodies. The majority of the antibodies were originally isolated from cohorts of individuals infected with SARS-CoV-2 in the first half of 2020 and were initially characterized by the Crowe lab (Zost et al., 2020), Nussenzweig lab (Robbiani et al., 2020), or Vir Biotechnology (Piccoli et al., 2020), with a few additional antibodies coming from commercial synthesis based on previously reported sequences (Jones et al., 2021; Hansen et al., 2020; Shi et al., 2020). The full deep mutational scanning data for all these antibodies are interactively displayed at https://jbloomlab.github.io/SARS2_RBD_Ab_escape_maps/ (last accessed 11 March 2022) and available in raw form at https://raw.githubusercontent.com/jbloomlab/SARS2_RBD_Ab_escape_maps/main/processed_data/escape_data.csv (see https://github.com/jbloomlab/SARS2_RBD_Ab_escape_maps/blob/bioRxiv_v1/processed_data/escape_data.csv for a stable version of the raw data corresponding to that used in this paper) (last accessed 11 March 2022).

The deep mutational scanning measures an escape fraction for each tolerated RBD mutation against each antibody, which represents an estimate of how completely that mutation escapes antibody binding (Greaney et al., 2021c). We summarize the mutation-level escape fractions into site-level measurements in two ways: taking the sum of the mutation escape fractions at each site or taking the mean of the mutation escape fractions across all tolerated mutations at each site. The results reported in this paper use the sums as the site-level metric, although the estimator has an option (escape_metric) to use the mean instead (the results are almost identical: compare Fig. 3 versus Supplementary Fig. S1).

We normalize the site-level escape metrics for each antibody to account for different strengths of antibody selection in different experiments using the approach described in Greaney et al. (2021a) and implemented in https://jbloomlab.github.io/dmslogo/dmslogo.utils.html#dmslogo.utils.AxLimSetter (last accessed 11 March 2022) with min_upperlim=1 and max_from_quantile=(0.5, 0.05): essentially this corresponds to scaling the site-escape values for each antibody so that a value of one corresponds to the larger of the maximum escape at a site or twenty times the median value across sites. Specifically, the normalization is done so that for each antibody *a*, the maximum escape $x_{a,r}$ at any site *r* is set so that $1 = \max\left(\max_r \left[x_{a,r}\right], 20\tilde{x}_{a,r} \right)$ where $\tilde{x}_{a,r}$ is the median of $x_{a,r}$ across sites *r* for antibody *a*. The rationale for this normalization is that it usually scales the site-level escape metric so that the maximum value at a site for each antibody is one, but for very ‘flat’ escape profiles where no site has more escape than twenty times the median, then the maximum value is smaller corresponding to no real peaks of escape for the antibody.

### 3.4. Estimation of the impact of mutations

The escape estimator determines the impact of mutating sites by calculating how much each antibody is escaped by mutations at each site and adjusting its contribution to the overall polyclonal mix accordingly.

Specifically, for each Antibody *a* we have a deep mutational scanning measurement $x_{a,r}$ of how much mutating *r* escapes that antibody. In the absence of any mutations, the overall escape map shown for the polyclonal mix is simply the mean over all antibodies, $\frac{1}{A}\sum_a x_{a,r}$ where *A* is the number of antibodies.

Let $\mathcal{M}$ be the set of sites that are mutated. Then for each antibody we compute the binding retained as $b_a\left(\mathcal{M}\right) = \left(\prod\limits_{r \in \mathcal{M}} \frac{\max_{r^{\prime}}\left(x_{a,r^{\prime}}\right) - x_{a,r}}{\max_{r^{\prime}}\left(x_{a,r^{\prime}}\right)}\right)^s$. Essentially, this equation means that if the RBD is mutated at a strong site of escape for an Antibody *a*, much of the binding is lost (if it is the strongest site of escape, all binding is lost). The variable *s* represents how dramatically binding is lost for mutations at sites of escape that are not the strongest: larger values of *s* means mutations at even moderate sites of escape reduce binding a lot. In this paper we report calculations with *s* = 2, although the estimator has an option (mutation_escape_strength) to choose other values. We then define the escape map after the mutations $\mathcal{M}$ as $\frac{1}{A}\sum_a x_{a,r} b_a\left(\mathcal{M}\right)$. The estimator shows the escape map with no mutations in gray and that after mutations in blue.

The overall antibody binding scores represent the fraction of antibodies that still bind and are calculated simply as $\frac{1}{A}\sum_a b_a\left(\mathcal{M}\right)$.

### 3.5. Implementation of interactive estimator

The interactive estimator at https://jbloomlab.github.io/SARS2_RBD_Ab_escape_maps/escape-calc/ (last accessed 11 March 2022) is implemented using Altair (VanderPlas et al., 2018), which is in turn built upon Vega-Lite (Satyanarayan et al., 2017).

### 3.6. Python module with batch-mode estimator

A Python module that implements the calculations is at https://github.com/jbloomlab/SARS2_RBD_Ab_escape_maps/ (last accessed 11 March 2022) and has all the same options as the interactive estimator.

### 3.7. Compilation of neutralization titers from the literature

For Fig. 3, we compiled neutralization data from three published studies on SARS-CoV-2 variants and mutants (Lucas et al., 2021; Uriu et al., 2021; Wang et al., 2021). For each study cohort, we computed the geometric mean fold change in neutralization titer over all subjects. The numerical compiled data are at https://github.com/jbloomlab/RBD_escape_calculator_paper/tree/main/results/neut_studies (last accessed 11 March 2022).

### 3.8. Mutations in SARS-CoV-2 variants

For Fig. 4, the definitions of which RBD sites are mutated in each variant are shown in .

## Supplementary Material

veac021_Supp
