# Supplementary material for: An antibody-escape estimator for mutations to the SARS-CoV-2 receptor-binding domain
Source: Virus Evol. 2022 May 11;8(1):veac021. doi: 10.1093/ve/veac021 (PMC9092643; doi:10.1093/ve/veac021)
Supplement: veac021_Supp [file veac021_supp.zip › supp.pdf]

## Supplementary Material

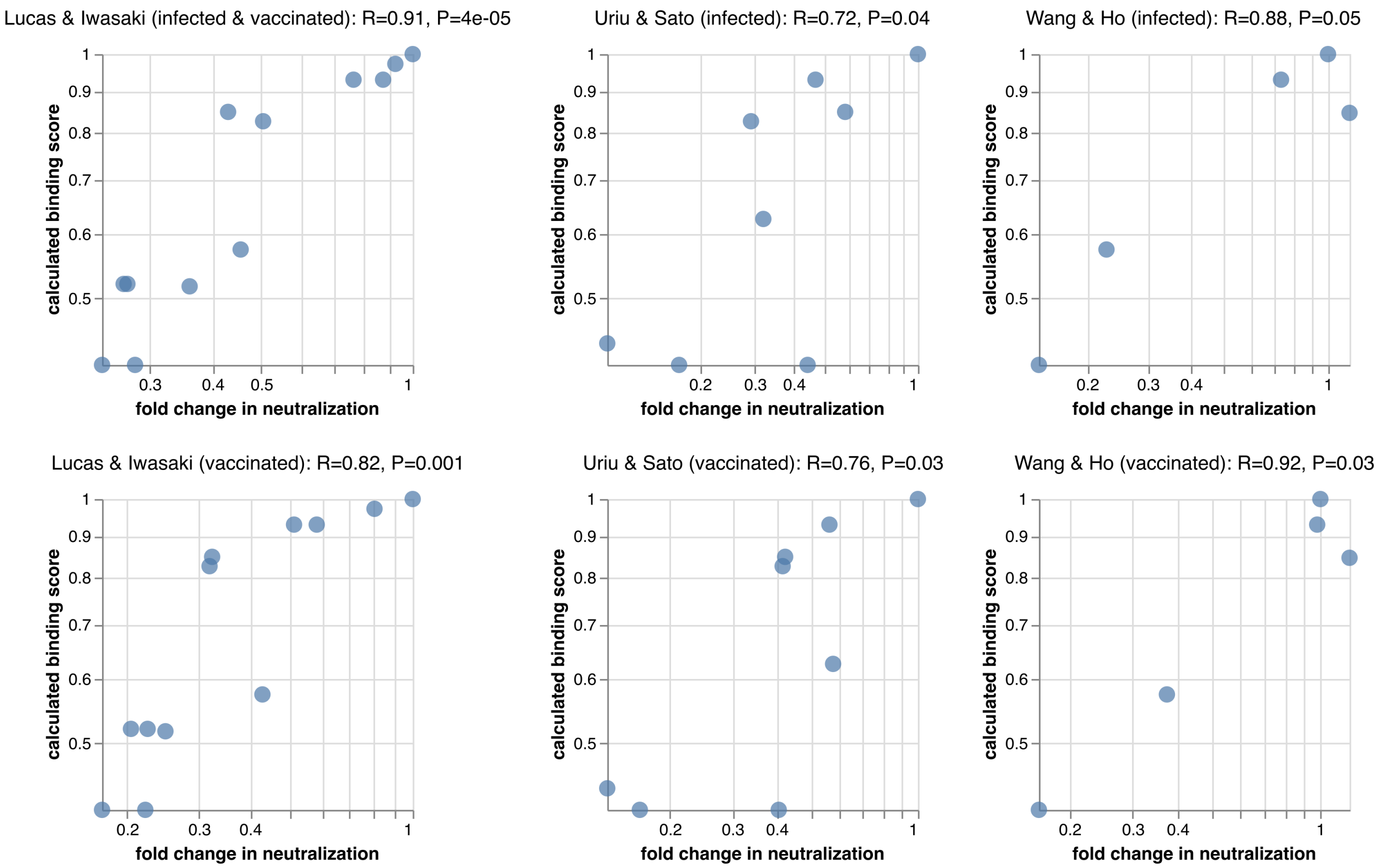


**Figure S1** A version of Figure 3 where the estimations are made using the mean effects of tolerated mutations at a site rather than the total (sum) of the effects of tolerated mutations at a site. The results are almost identical.
